# School-based intervention that integrates nutrition education and supportive healthy school food environment among Malaysian primary school children: a study protocol

**DOI:** 10.1186/s12889-019-7708-y

**Published:** 2019-10-30

**Authors:** Choon Huey Teo, Yit Siew Chin, Poh Ying Lim, Shahril Azian Haji Masrom, Zalilah Mohd Shariff

**Affiliations:** 1 Department of Nutrition & Dietetics, Faculty of Medicine & Health Sciences, Universiti Putra Malaysia, UPM, 43400 Serdang, Selangor Malaysia; 2 Department of Nutrition, Batu Pahat District Health Office, 83000 Batu Pahat, Johor Malaysia; 3 Research Centre of Excellence, Nutrition and Non-Communicable Diseases, Faculty of Medicine and Health Sciences, Universiti Putra Malaysia, UPM, 43400 Serdang, Selangor Malaysia; 4 Department of Community Health, Faculty of Medicine & Health Sciences, Universiti Putra Malaysia, UPM, 43400 Serdang, Selangor Malaysia; 5 Department of District Health Office, Batu Pahat District Health Office, 83000 Batu Pahat, Johor Malaysia

**Keywords:** Children, Malnutrition, School-based intervention, School nutrition, Nutrition education, School food environment

## Abstract

**Background:**

Malnutrition among school children may contribute to adverse health consequences such as non-communicable diseases, poor cognitive performance, psychological distress and poor quality of life that may persist into adulthood. In order to prevent childhood malnutrition, an intervention programme that integrates nutrition education and healthy school food environment is needed to provide nutrition information and reinforce the skills on healthy eating behaviours in schools. This paper describes a study protocol of a school-based intervention programme that integrates nutrition education and healthy school food environment, namely School Nutrition Programme (SNP). The SNP is a primary prevention programme that promotes healthy lifestyle among primary school children in light of the high prevalence of malnutrition in Malaysian children.

**Methods/design:**

This quasi-experimental study aimed to evaluate the effectiveness of the SNP between intervention and comparison groups before and after the SNP, and after a 3-month follow-up. The SNP consisted of two main components, whereby three nutrition education sessions were implemented by trained teachers using three standardised modules, and healthy school food environment was implemented by the canteen food handlers with the provision of healthy menu to children during school recess times. Children from intervention group participated in the SNP, in addition to the standard Physical and Health Curriculum. The comparison group attended only the standardised Physical and Health Curriculum and the school canteen food handlers were reminded to follow the standard canteen guidelines from the Ministry of Education Malaysia. The assessment parameters in evaluating the effectiveness of the programme were knowledge, attitude and practice on nutrition, eating behaviours, physical activity, body composition, psychological distress, cognitive performance and health-related quality of life. Assessments were conducted at three time points: pre-intervention, post-intervention and 3-month follow-up.

**Discussion:**

It was hypothesised that the SNP would be effective in promoting healthy lifestyle among school children, and further contributes in preventing malnutrition problem, enhancing cognitive performance and improving health-related quality of life among school children. Findings of the present study can be expanded to other schools in future on ways to improve nutrition education and healthy school food environment.

**Trial registration:**

UMIN Clinical Trial Registration UMIN000032914 (Date of registration: 7th June 2018, retrospectively registered).

**Protocol version:**

16th September 2019 & Version 4

## Background

The World Health Organization reported that 151 million and 51 million children under 5 years of age were stunted and wasted respectively; on the other hand, the number of overweight or obese children under the age of five was 38 million in 2017 globally [1]. More than half of stunted children, more than two thirds of wasted children and almost half of overweight children lived in Asia [2]. In South-East Asia, at least one in every four children under five was stunted; at least one in every 10 children under five was wasted and overweight respectively [2].

Overnutrition and undernutrition coexist among primary school children in Malaysia [3–6]. The National Health and Morbidity Survey (NHMS) 2017 showed that the prevalence of overnutrition (overweight 16.3%; obesity 17.4%) were two times higher than undernutrition (thinness 6.7%; stunting 7.8%) among Malaysian children aged 10 to 12 years [7]. In addition, the prevalence of overnutrition among primary school children (33.7%) was higher than secondary school adolescents (28.5%) [7]. Malnutrition refers to deficiencies, excesses or imbalances in a person’s intake of energy and/or nutrients [8]. Undernutrition may increase risks of impaired physical and cognitive performance, as well as morbidity and mortality during childhood [9, 10]. On the other hand, overnutrition is associated with increased risks of psychological distress such as depression, anxiety and social withdrawal [11–13]; low self-esteem, poor quality of life and cognitive deficits, especially in executive function [14, 15]. In order to achieve the Sustainable Development Goals, it is important to recognise the role of prevention and health promotion in order to reduce the prevalence and the burden of diseases [16]. Therefore, designing an effective healthy lifestyle programme that is interconnected with the education programmes in primary school can improve health literacy that is needed to prevent this public health concern among primary school children.

In Malaysia, children aged 7 to 12 years old are generally enrolled in primary school levels for 6 years, which is known as Standards 1 to 6 [17]. Lifelong nutritional patterns are formed during childhood, which can reach at influential stages in their lives [18, 19]. Previous studies reported that Malaysian primary school children are at increased risk of poor dietary behaviours, including breakfast skipping, low fruits and vegetables intakes, unhealthy snacking behaviours and low physical activity [7, 20, 21], which may affect their nutritional status and expose children to malnutrition [22, 23], lower cognitive performance and poor quality of life [24–26]. There is a need to have a holistic nutrition intervention programme that promotes healthy eating and active living for all primary school children, so that they can have a healthy lifestyle, good nutritional status, better cognitive performance and good quality of life.

This paper describes the protocol of School Nutrition Programme (SNP) that consisted of two main components, namely nutrition education and healthy school food environment. The present study aimed to evaluate the effectiveness of SNP on differences in primary outcome measures including anthropometric measurements, knowledge, attitude and practice on nutrition, eating behaviours, physical activity; and secondary outcome measures including psychological distress, cognitive performance and health-related quality of life among primary school children. A process evaluation was focused on the attendance rate of children during the School Nutrition Campaign, teachers and canteen food handlers during training of trainers; and the programme feedbacks from children, teachers and canteen food handlers after the study.

## Methods/design

### Study design

The present study design was a quasi-experimental design. Out of 147 National Primary Schools in Batu Pahat district, a total of 143 National Schools met the inclusion criteria of being co-educational, non-religious, non-special educational, multi-ethnic and government funded schools. Then, these 143 schools were stratified based on the three main school types, namely i) National Primary School (SK), ii) National Chinese Primary School (SJKC) and iii) National Tamil Primary School (SJKT). Two schools from each stratified group were selected. One school was selected as Intervention Group (IG) and one as Comparison Group (CG) from each school type. All children from these selected schools were invited to participate in the study.

### Participants

**Fig. 1:**
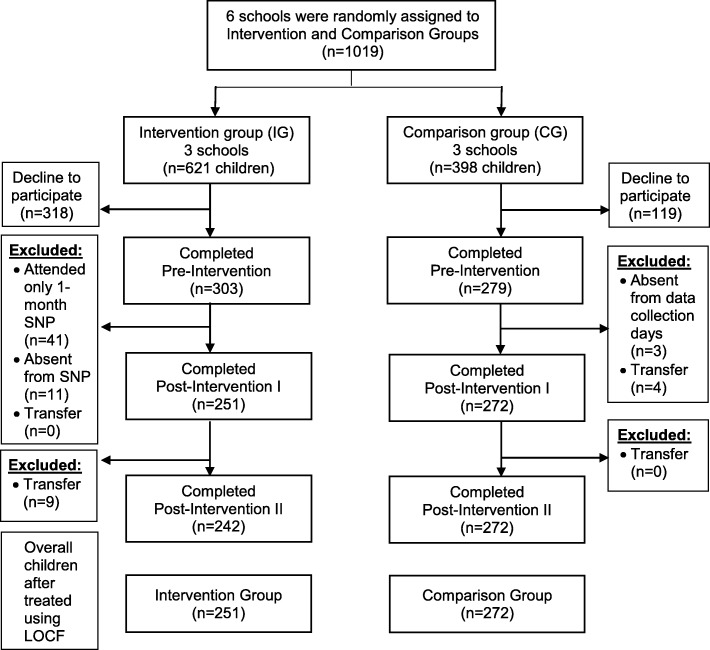
Children’s flow diagram

This study was conducted in six selected schools in Batu Pahat District, Johor State, Malaysia. School authorities (principal and teachers) and canteen food handlers from three selected intervention schools were invited to participate in the study. All Malaysian children (males and females) who were enrolled in Standards 1 to 5 (aged 7 to 11 years) and who was either Malay, Chinese or Indian were invited in the study (Fig. 1). Upon request from the Ministry of Education (MOE) Malaysia, children in Standard 6, who would be attending the National Primary School Evaluation Test, were excluded from the study. In addition, children with learning disorders, obvious genetic disorders such as attention deficit hyperactivity disorder (ADHD) were excluded from the study. The children were assessed prior to the start of the programme (Pre-Intervention), one-week right after completing SNP (Post-Intervention I) and three-month follow-up without intervention (Post-Intervention II).

### Sample size calculation

The sample size for primary school children was calculated using Aday’s (2006) equation [27]. Based on a previous study in Malaysia, the mean difference of waist circumference between intervention and comparison group after the intervention programme was taken into consideration to calculate the sample size of the SNP [28].

$$ \mathrm{n}=\frac{2{\upsigma}^2{\left[{\mathrm{Z}}_{1\hbox{-} \upsigma /2}+{\mathrm{Z}}_{1\hbox{-} \upbeta}\right]}^2}{{\left({\upmu}_1\hbox{-} {\upmu}_2\right)}^2} $$

Based on the study, the mean change in waist circumference for intervention group after the intervention programme was 0.1 cm, whereas mean change in waist circumference for comparison group after the intervention programme was 2.2 cm. The calculated sample size was 195 children in intervention and comparison groups, respectively. The sample size was also adjusted for a response rate of 80.0%. The intended sample size was 488 primary school children from Standards 1 to 5 in Batu Pahat District. Considering a power of 80% and a level of significance of 5%, a total of 244 children was needed for each intervention and comparison group, respectively. The small effect size of 0.09 was obtained.

### Description of School Nutrition Programme intervention

The SNP aimed to prevent malnutrition among primary school children. The rationale of SNP was to increase the children’s nutrition and physical activity knowledge, attitude and practices to optimise their healthy lifestyle by encouraging them to eat healthily and stay physically active. The SNP is based on the Social Cognitive Theory (SCT) [29], which emphasises the importance of social and environmental factors in determining the psychosocial and behavioural risk factors of both undernutrition and overnutrition. The SNP is composed of two main components: (1) delivering nutrition education through School Nutrition Campaign for 3 months; and (2) serving healthy menu during school recess time to the children over a period of 3 months. The children of the intervention group participated in three School Nutrition Campaigns, in addition to the standard Physical dan Health Curriculum. The children in the comparison group received standard Physical and Health Curriculum and school canteen food handler were reminded to follow canteen guidelines by MOE Malaysia.

For nutrition education component, the researchers conducted three School Nutrition Campaigns using the three different nutrition education modules and education materials of Healthy Kids Programme [30]. These nutrition education modules included four main aspects, namely health awareness, nutrition, physical activity and hygiene. Specifically, module 1 comprised six topics with the goal of educating primary school children on the importance of healthy lifestyle and introduce the concept of food pyramid, physical activity and personal hygiene. Module 2 comprised six topics with the goal to provide more in-depth knowledge on food groups in the Malaysian Food Pyramid and Physical Activity Pyramid. Module 3 comprised five topics with the goal to strengthen the important skills to apply the knowledge that the children learnt from module 1 and module 2. These modules incorporated the “fun while learning” concept, with various attractive engagement activities such as act and guess, role-play, quiz and detective games. There was a four-week gap between each campaign, with at least 6 hours per campaign.

The training of trainers (TOT) aimed to provide hands-on training to the teachers to carry out the nutrition education component of the SNP, and to empower them to convey healthy lifestyle messages to the children during the School Nutrition Campaigns. As there were three modules, three separate sessions of the TOTs (1 day training session for each TOT) were needed to be completed by the teachers. The trained teachers conducted the nutrition education sessions to the children during the School Nutrition Campaigns and each topic took approximately 1 hour to deliver.

The second component of the SNP is a supportive healthy school food environment, which aimed to increase the availability of healthy foods in primary school canteen and to provide training to the canteen food handlers. A 1-day TOT of canteen food handlers was conducted to all food handlers by using a revised Healthy Catering Module from the Nutrition Division, MOH Malaysia [31], with incorporation of a few interactive games during the training. The food handlers learned to serve healthier foods, the fundamentals of preparing healthy foods, recipe modification, menu planning and healthy cooking techniques. All canteen food handlers needed to fulfil the basic requirements from the MOE Malaysia, which were to attend the Food Handling Course and compulsory get anti-typhoid vaccination [32].

During the TOT of canteen food handlers, the canteen food handlers discussed and planned a one-month healthy menu together with the researchers. One-week after the TOT of canteen food handlers, all canteen food handlers prepared four menus assigned by the researchers during Menu Testing Day. Stakeholders (such as principal, student affair teacher, Parent Teacher Association and school children) were invited to taste the healthy menu. Their comments and feedbacks were noted and used to further improve the menu. The healthy menu should contain a variety of food groups, such as from rice, noodle or bread, vegetable, fruit, and meat or chicken. Local food availability and children’s acceptability were taken into consideration when planning for the healthy menu. All menus were monitored by researchers and the trained teachers during the intervention period. Therefore, students would be able to practise healthy eating during school recess. Throughout the study period, the researchers would inform and remind the children about the importance of programme adherence.

### Measurements

All participants in both intervention and comparison groups were assessed prior to the start of the programme (Pre-Intervention), one-week right after completing the School Nutrition Campaigns (Post-Intervention I) and three-month afterwards without intervention (Post-Intervention II). The questionnaires were prepared in Malay, Chinese and Tamil languages to ease the children in understanding the questionnaires better. The anthropometric measurements and cognitive assessments of the children were conducted by trained researchers.

#### Socio-demographic background

In the present study, socio-demographic background of children in both intervention and comparison groups were determined. Children answered the items regarding their socio-demographic background, such as sex, ethnicity, age and date of birth, while parents answered questionnaires regarding parental education level and monthly household income.

### Impact evaluation

Impact evaluation included primary outcome measures, namely anthropometric measurements, knowledge, attitude and practice on nutrition, eating behaviours, physical activity; and secondary outcome measures included psychological distress, cognitive performance and health-related quality of life.

### Primary outcomes

#### Anthropometric measurements

Body weight of the children was measured using weighing scale and recorded to the nearest 0.1 kg. Height of the children was measured using stadiometer and recorded to the nearest 0.1 cm. Body weight and height were used for BMI calculation using the BMI formula BMI = weight (kg) / height^2^ (m^2^) [33]. The z-scores of weight-for-age (WAZ), height-for-age (HAZ) and BMI-for-age (BAZ) were computed using WHO AnthroPlus software [34], the nutritional status of children was obtained by comparing the z-scores against the WHO Growth Reference 2007 tables [33]. Waist circumference was measured by using Lufkin Executive diameter steel tape Model W606 PM. Abdominal obesity was determined by referring to the 90th percentile cut-off-point of waist circumference for children aged 7–11 years [3]. Percentage of body fat for children aged 10–11 years was measured using Omron HBF-306 Body Fat Analyzer Scale (Omron Corporation, Japan). Based on the obtained body fat percentage, the children were categorised into normal and high body fat percentage [35]. All assessments by qualified researchers followed the International Society for the Advancement of Kinanthropometry (ISAK) procedures.

#### Knowledge, attitude and practices on nutrition

Knowledge, attitude and practices on nutrition of children were measured by using the knowledge, attitude and practices on the modules of Healthy Kids Programme [30]. The questionnaire consisted 30 knowledge items in the format of multiple choices. Each knowledge on nutrition item had four answer options. Each correct response was allocated one point and an incorrect answer was allocated 0 point. The possible range of scores for knowledge was between 0 and 30. A higher score indicated a higher level of knowledge on nutrition. Besides, there were 30 attitude items. Each attitude item was measured on a 3-point Likert scale; from agree (3 points) to disagree (1 point). The possible range of scores for attitude was between 10 and 30. The total attitude score was summed up and a higher attitude score indicated better attitude on nutrition. In addition, there were a total of 30 practice items. The practice items were assessed on a 5 point-frequency scale, ranging from almost every day [5] to never [1]. The possible range of scores for practice was 10–50. All the frequency points were summed up, and a higher score indicated healthier practice on nutrition by the children.

#### Eating Behaviours

Eating behaviours of the children, which included main meal consumption and snacking behaviour were assessed based on the Eating Behaviours Questionnaire (EBQ) [36]. This questionnaire was designed to measure the frequency of main meal consumption (breakfast, lunch and dinner) and frequency of snacking between meals (morning tea break, evening tea break and supper). The frequency of meal consumption was based on number of days consumed in a week [36]. For snacking behaviour among the children, the number of days that the children consumed morning tea, afternoon tea and supper in a week were asked.

#### Physical activity

Physical Activity Questionnaire for Children (PAQ-C) is appropriate for elementary school-aged children (approximately ages 8–14 years) who are currently in the school system and have recess as a regular part of their school week [37, 38]. The PAQ-C is a self-administered, 7-day recall instrument, which provides a summary physical activity score derived from nine items, each scored on a 5-point scale. Item 1 (spare time activity) from no activity = 1; 7 times or more = 5. Items 2 to 8 (PE, recess, lunch, right after school, evening, weekends) from lowest activity response = 1 or highest activity response = 5. Item 9 (mean of all days of the week) from none = 1; very often = 5. Item 10 (identifies students who are unusual active during the previous week). By adding up all means of the first nine items in PAQ-C, a summative score of physical activity was obtained. A score of 1 indicated low physical activity level, whereas a score of 5 indicated high physical activity level. The Cronbach’s alpha of PAQ-C was 0.89 and it was higher than the acceptance level (0.60) [37].

### Secondary outcomes

#### Psychological distress

The Revised Child Anxiety and Depression Scale (RCADS-30) was used to measure symptoms of psychological distress, namely separation anxiety disorder, social phobia, generalised anxiety disorder, panic disorder, obsessive compulsive disorder, and major depressive disorder [39]. A 30-item version of RCADS (RCADS-30) was developed whereby this briefer version has five to six items per subscale. A self-report scale was used with subscale corresponding to separation anxiety disorder (5 items), social phobia (5 items), generalised anxiety disorder (5 items), panic disorder (5 items), obsessive compulsive disorder (5 items) and major depressive disorder (5 items). Items were scored 0–3 corresponding to never, sometimes, often and always respectively. A higher score indicated having more severe anxiety and depression symptoms [40]. The internal consistency of the RCADS-30 was tested and the Cronbach’s alpha for total scale was 0.89, with alpha coefficients for the separate RCADS-30 subscales ranging from 0.68 (obsessive-compulsive disorder) to 0.78 (generalised anxiety disorder) [40].

#### Cognitive performance

Raven’s Coloured Progressive Matrices (CPM) was used to assess cognitive performance of children [41]. This test contained 36 items with three sets of 12 items in each (sets A, AB and B) from the standard matrices. Most items were presented on a coloured background to make the test visually stimulating for participants. Each child needed around 15 min to complete the test. Children were asked to find and complete the missing pattern. Each correct answer was given 1 mark. The raw score on the coloured progressive matrices test ranged between 0 and 3. The child’s mental age is thus the age at which the median score is equal to his or her raw score. This score was converted into a standard score based on the Raven’s CPM norm table. A higher score indicated better cognitive performance.

#### Health-related quality of life

Pediatric Quality of Life Inventory 4.0 (PedsQL 4.0) was used to assess health-related quality of life (HRQoL) among children aged 8–12 years [42]. There was a total of 23 items from 4 subscales for child report: My health and activities; My feelings, I can get along with others and About school. Children were asked to recall health-related problems in the past 1 month and rate each item on a five-point Likert scale (0 = never, 1 = almost never, 2 = sometimes, 3 = often, 4 = almost always). Scores of all items were plotted on a 0-100 point scale (0 = 100, 1 = 75, 2 = 50, 3 = 25, 4 = 0). Higher total PedsQL scores corresponded to better HRQoL. The Cronbach’s alpha of PedsQL was 0.89 and it was higher than the acceptance level (0.60) [43].

### Process evaluation

Process evaluation of the SNP consisted of evaluation of attendance rate and programme feedback among the intervention groups. The attendance rate was assessed by using the attendance list for implementation of School Nutrition Campaigns 1, 2 and 3, respectively. Teachers needed to use the attendance list prepared by the researchers and took the attendance of children for each camp. Overall attendance rate and attendance by each camp were calculated. Programme feedback forms for three camps were self-administered by children after completing each of the campaigns. Percentage of programme feedback forms submitted by each camp was calculated. The attendance rate and programme feedback of TOT for teachers and TOT for canteen food handlers were also assessed in this study.

### Data analysis

The evaluation of the study was based on the modified intention-to-treat analysis to retain children in the study [44]. Children who fulfilled the following criteria would be included in the data analysis process:

i. Children of the intervention group who have attended at least two camps in the School Nutrition Programme
ii. Children from both the intervention and comparison groups who had completed Pre-Intervention, Post-Intervention I and Post-Intervention II.

All statistical analysis will be conducted using IBM SPSS statistics 24.0. Descriptive analysis such as frequency and percentages, will be reported for categorical variables, while means and 95% confident interval will be reported for continuous variables. Skewness test of normality will be used to assess the normality of continuous variables. A value of skewness within the range of ±2.0 will be considered as normally distributed [45]. The Chi-square Test or Fisher’s Exact Test will be used to determine the association between two or more groups of categorical variables. The Independent sample t-test will be used to compare the mean values of the continuous variables between intervention and comparison groups and Paired sample t-test will be used to compare the mean values at pre-intervention and post-intervention within the intervention and comparison groups.

Simple linear mixed model will be conducted to determine the association of each covariate (such as time, group, child’s age, parental education level and school type) with all the dependent variables (namely knowledge, attitude and practice on nutrition, eating behaviour, physical activity, anthropometric measurement, psychological distress, cognitive performance and health-related quality of life). Multiple linear mixed model will be conducted to investigate the dependent variables between groups (intervention and comparison) over time, adjusted for covariates. Interaction term of time and group will be included in the model. The differences in all continuous variables between intervention and comparison groups will be determined at three time points (Pre-Intervention, Post-Intervention I and Post-Intervention II). The statistical significance will be set at *p* < 0.05. The data will be stored for future use after the completion of the study.

### Ethical approval

Ethical approval for this study was obtained from the National Medical Research Registry (NMRR) and Medical Research Ethics Committee (MREC) of Ministry of Health (MOH), Malaysia (NMRR-17-1273-36,187 IIR). Permissions for field data collection in primary schools were obtained from MOE Malaysia, State Department of Education of Johor, District Education Centre of Batu Pahat and selected schools before conducting the study. The researchers explained the study protocol to the children and their parents by using the information sheet. Prior to data collection, written consents were obtained from children, their parents/guardians, school authorities (principal and teachers), and canteen food handlers, respectively.

## Discussion

The present study will evaluate the effectiveness of a school-based intervention to prevent malnutrition among Malaysian primary school children. The three-month SNP that integrated nutrition education and healthy school food environment aimed to improve knowledge, attitude and practice on nutrition, eating behaviours, physical activity, cognitive performance and health-related quality of life; and to reduce BMI-for-age among primary school children. In order to achieve the primary outcomes of the present study, Koo and colleagues (2018) showed that a 12-week nutrition education intervention was enough to significantly increase practice of wholegrain foods consumption on a daily basis, lower body mass index-for-age z-score, body fat percentage and waist circumference among the intervention group, which was in line with previous study [46].

In Malaysia, several nutrition intervention programmes have been carried out among children aged 8 years [47, 48], adolescents [49] and children with overweight and obesity problem [50], which reported effectiveness of nutrition education intervention programmes in improving knowledge, attitude and practices on nutrition. The efforts were mainly for nutrition education promotion on healthy eating and physical activity. For instance, a 3-week Nutrition Education Programme among 8-year-old children in Malaysia aimed to improve nutrition knowledge, attitude, and practice found that knowledge and attitude had improved at post-intervention and 6-months follow-up, but practice score did not increase throughout the study in the intervention group over time [47]. Another Nutrition Education Programme by Zalilah et al. [28] focused among 8-year-old children showed that there were positive impacts on nutrition knowledge, attitude and practice after the intervention, but no follow-up result was reported after the programme. Zalilah et al. suggested that healthy family environment, healthy food availability and accessibility in schools should be included in nutrition intervention in order to sustain nutrition practices [48]. Tee et al. [31] also recommended that a successful nutrition intervention should include content and teaching strategies that are developmentally appropriate for the children and address changes in the environment. To date, there is no published study that focuses on promoting healthy school food environment in Malaysia. Therefore, it is important to develop an intervention that integrates nutrition education and school food environment, especially the school canteen that can exert a strong influence on children’s food decisions.

In Malaysia, primary school children consume their morning tea break during recess at school canteens, some children would take their lunch at school when they have tutorial classes or co-curricular activities. The Healthy School Canteen Guideline was developed and implemented by MOE Malaysia since year 2011. These guidelines aimed to improve the food service of school canteens and cultivate a balanced and healthy food intake in schools [32]. Based on the assessments of 113 primary school canteens in Batu Pahat district conducted in the year 2015, about one-third (33.6%) of the canteens were under the “Dissatisfactory” category, indicating non-compliance to the guidelines [51]. Most of the canteen caterers sold cordial drinks, carbonated drinks and fried foods especially processed foods like nugget, burger and hotdog; while insufficient green vegetables and fruits were prepared for the students [51]. Frederike et al. reported that the nutritional intake among United States school children was significantly affected by the school food environment because children spend many hours at school each day [52, 53]. A systematic review and meta-analysis stated the effectiveness of healthy school food environment in increasing the consumption of fruits and vegetables, while reducing total fat intake, saturated fat intake and sodium intake [54]. In order to increase healthy food availability in primary school canteens, it is important to address the concerns and knowledge of canteen food handlers [55]. However, Lessa et al. found that food handlers had a significantly lower food nutrition knowledge score than the general public [56]. Therefore, an integrate intervention, which includes collaboration between canteen food handlers and nutritionist is needed to ensure that appealing, healthy menu is prepared for the primary school children.

In Japan, besides serving nutritious food, the school lunch programme namely *Shokuiku* also serves as an important nutrition educational approach for school children to acquire proper nutrition knowledge, and to impart a sense of gratitude in children [57]. To the best of our knowledge, the present study is the first school-based nutrition intervention that integrates nutrition education and healthy school food environment among primary school children in Malaysia. This School Nutrition Programme is hypothesised to improve knowledge, attitude and practices on nutrition, eating behaviours, physical activity, anthropometric assessments, psychological distress, cognitive performance and health-related quality of life among Malaysia primary school children. Therefore, the examination on the effectiveness of a successful school nutrition approach within school setting is of great importance.

The sustainability of the intervention beyond the study duration will be considered through training of school teachers on the nutrition education curriculum and the transformation of school canteens by preparing a variety of nutritious menu for children during school recess. The World Health Organization reported that implementation of a comprehensive programme that promotes healthy school food environment, health and nutrition education and physical activity among school-age children and adolescents can end childhood malnutrition issues [1, 28]. Establishment of standards for meals provided in schools by increasing the availability of whole grains foods sold in schools that meet healthy nutrition guidelines and eliminate the provision or sales of unhealthy foods, such as sugar sweetened beverages and energy-dense, nutrient-poor foods, in the school environment are important. Previous local studies suggested that modifiable dietary habit strategies such as increasing fruits and vegetables intake, increasing whole grains intake, limiting consumption of sweetened drinks and controlling portion sizes were needed to combat childhood obesity [58, 59]. Importance of proper diets has been recognised not only for the control of lifestyle-related diseases, but more so in various physical aspects to improve the quality of life, including good growth of children [60].

Findings of the current study can be used by future researchers, health professionals, policy makers and school authorities to plan and implement the intervention in preventing malnutrition among primary school children in Malaysia. Furthermore, policy makers (including Parent Teacher Association), health agencies, programme planners and community leaders can use the data obtained from this study for planning and implementing effective policies as well as other intervention programmes that consist of nutrition education and healthy school food environment for Malaysian children to promote healthy lifestyle. The current study is important as it may raise awareness of the public, especially school teachers and canteen food handlers to give more attention towards the importance of nutrition education and healthy menu in canteens to Malaysian primary school children. This intervention study is in line with the National Plan of Action for Nutrition of Malaysia [NPANM] III (2016–2025), where nutrition education promotion is needed in schools and School Meal Programme has to be implemented to at least three schools by the year 2020 and at least six schools by the year 2025 in every state in Malaysia [61].

## Data Availability

Data sharing is not applicable to this article as no datasets were generated or analysed during the current study.

## Abbreviations

BMI: Body Mass Index
ISAK: International Society for the Advancement of Kinanthropometry
MOE: Ministry of Education
MOH: Ministry of Health
NHMS: National Health and Morbidity Survey
PTA: Parent Teacher Association
SNP: School Nutrition Programme
WHO: World Health Organization
